# Successful endoscopic removal of foreign body lacerating into the duodenum

**DOI:** 10.1016/j.vgie.2022.07.014

**Published:** 2022-10-22

**Authors:** Hadiatou Barry, Rami Abusaleh, Lauren Mazin, Sandra Elmasry, Keng-Yu Chuang

**Affiliations:** 1 Department of Medicine, Creighton University, St. Joseph Medical Center; 2 Internal Medicine, Creighton University, St. Joseph Medical Center; 3 Department of Medicine, Creighton University, Valleywise Medical Center; 4 School of Medicine, Creighton University, St. Joseph Medical Center, Phoenix, Arizona; 5 Department of Medicine, Creighton University, Valleywise Medical Center; 6 Department of Medicine, Creighton University, Valleywise Medical Center

## Abstract

Video 1 The endoscope was advanced under direct visualization to the third part of the duodenum. A foreign body consistent with a ballpoint pen was identified in the duodenum. The sharp end of the pen formed a deep laceration through the second portion of the duodenum. The blunt end of the pen was ulcerated into the third portion of the duodenum with 2 additional pressure ulcers located in close proximity. Removal of the pen was first attempted using a snare without success. The foreign body was then successfully removed with a rat tooth. The second portion of the duodenum showed minimal oozing with contained deep laceration. The third portion of the duodenum showed 2 pressure ulcers.

The endoscope was advanced under direct visualization to the third part of the duodenum. A foreign body consistent with a ballpoint pen was identified in the duodenum. The sharp end of the pen formed a deep laceration through the second portion of the duodenum. The blunt end of the pen was ulcerated into the third portion of the duodenum with 2 additional pressure ulcers located in close proximity. Removal of the pen was first attempted using a snare without success. The foreign body was then successfully removed with a rat tooth. The second portion of the duodenum showed minimal oozing with contained deep laceration. The third portion of the duodenum showed 2 pressure ulcers.

## Case Report

**Figure 1:**
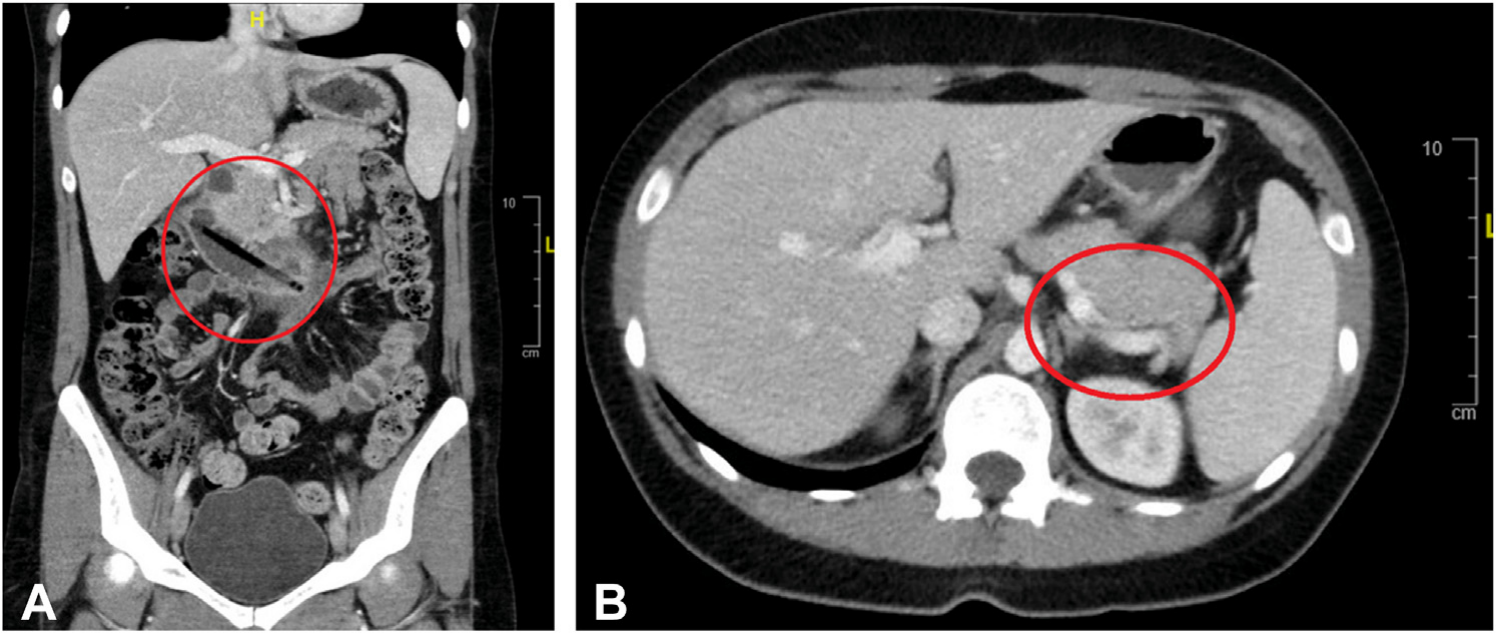
CT scan anteroposterior with intravenous contrast showed coronal section **(A)** and horizontal section **(B)** ingested “pen-like” 11- × 0.5-cm foreign body at the second and third portion of the duodenum. The foreign body deep laceration 2 cm through the second portion of the duodenum at the right lateral wall, adjacent to trace subhepatic and small pelvic free fluid. Additional duodenal wall thickening/inflammation at the third portion without additional deep laceration.

A 29-year-old woman with psychiatric history and multiple foreign body ingestions presented with a 1-day history of moderate to severe nonradiating central abdominal pain. She reported ingesting foreign objects 3 weeks prior to presentation. The patient was tachycardiac but afebrile and hemodynamically stable, and her abdominal examination showed right upper quadrant tenderness with negative Murphy sign. A CT scan showed a foreign body identified as a ballpoint pen laceration in the second part of the duodenum, projecting at the subhepatic region with adjacent free fluid but no free intraperitoneal air (Fig. 1). After a multidisciplinary discussion with surgical colleagues, the patient went to the operation room for an endoscopic attempt at foreign body removal with deep laceration closure if needed while surgeons were on standby.

**Figure 2:**
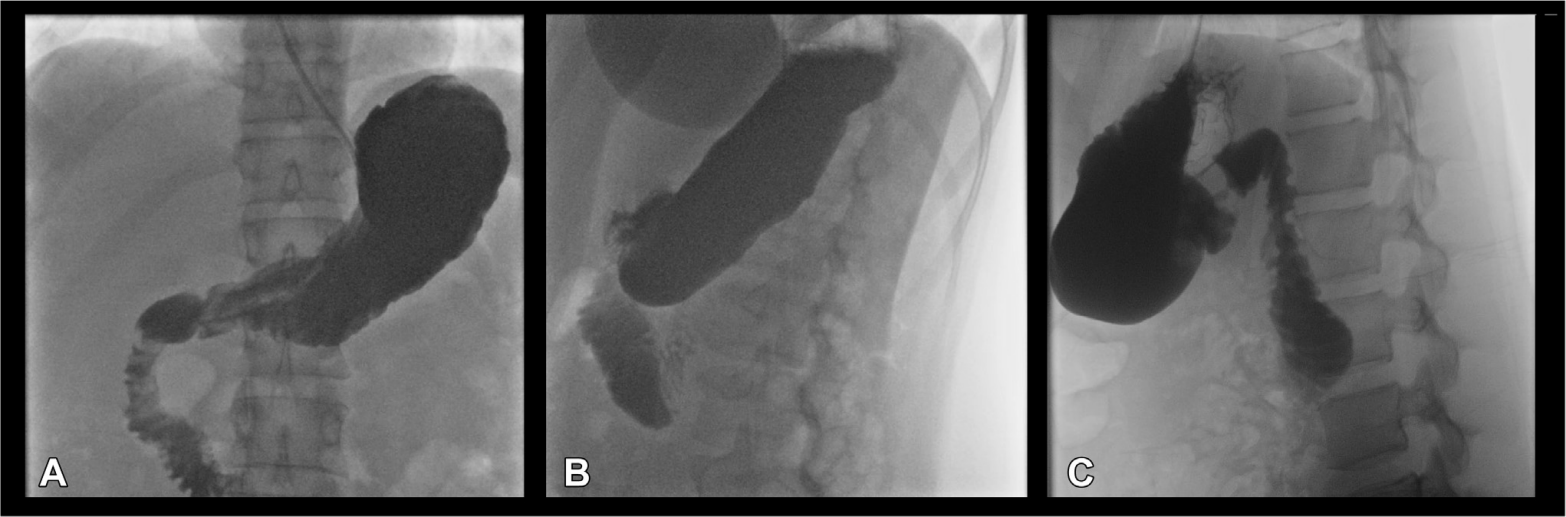
(**A**) Anterior (coronal view) abdominal X-ray showing the early phase of contrast with trace in the esophagus, with fall through and filling stomach fundus, body, pylorus, and duodenum, without an extraintestinal leak. (**B**) Lateral (sagittal view) abdomen X-ray showing contrast with later stage fall through and filling the lower part of stomach fundus, whole stomach body, pylorus, and duodenum, without an extraintestinal leak. (**C**) Lateral (sagittal view) abdomen X-ray showing a later stage as contrast falls through the lower part of the stomach body, pylorus, and duodenum, without an extraintestinal leak.

The patient had successful endoscopic removal of the foreign object from the duodenum and control of bleeding. There was a deep laceration seen in the second part of the duodenum and pressure ulcers seen in the third part of the duodenum (Video 1, available online at www.giejournal.org). After the endoscopic removal, upper GI tract radiography series with barium swallow was performed, which showed no leak (Fig. 2).

## Procedure

The patient received antibiotics before the procedure. After adequate sedation was achieved, the patient’s esophagus was intubated, and the endoscope was advanced under direct visualization to the third part of the duodenum. A foreign body consistent with a ballpoint pen was identified in the duodenum. The sharp end of the pen deep lacerated through the second portion of the duodenum. The blunt end of the pen was ulcerated into the third portion of the duodenum with 2 additional pressure ulcers located in close proximity. Removal of the pen was first attempted using a snare without success. The foreign body was then successfully removed with a rat tooth while capturing the pen near the end to reduce the risk of slipping.

The second portion of the duodenum showed minimal oozing with contained deep laceration. The third portion of the duodenum showed 2 pressure ulcers (Video 1).

Because the patient had a deep laceration with questionable perforation, we performed an x-ray examination of the esophagus, stomach, and the duodenum with a barium swallow, which showed no leak and in which no clip was required to be placed.

## Discussion

Foreign body ingestion is a commonly encountered condition by gastroenterologists. Most of the time, foreign bodies in the esophagus and stomach can be removed safely through endoscopy.^1^^,^^2^ On the other hand, if retained, small-bowel foreign bodies are frequently removed through surgery. Typically, foreign bodies tend to pass the entire small bowel if they are small enough to traverse through the pylorus; however, the risk of perforation is increased if they are retained. The current reported estimated rate of incidences of bowel perforation because of foreign bodies is about 1%.^3^ Therefore, laparoscopy with the primary suture of the intestine is the mainstay of diagnosis and treatment for these types of cases. Perhaps, patients who are stable without signs or symptoms of acute abdomen or peritonitis can safely undergo an endoscopic attempt at foreign body removal and endoscopic closure of perforation with a clip, OverStitch suture device (Apollo Endosurgery, Austin, Tex), or over-the-scope-clip.^4^^,^^5^ Endoscopic procedures tend to be safer, less invasive, have shorter hospital stays, and decrease the risk of mobility and mortality.^2^^,^^5^ Studies show strong evidence of endoscopic management of iatrogenic perforation, especially when recognized early; therefore, a similar approach can be applied in stable patients with foreign body perforations.^1^^,^^2^ There is always a role for surgical intervention in patients with peritonitis, acute abdomen, large perforations, or where endoscopic attempts fail.^5^

## Conclusion

This video demonstrated that endoscopic removal of foreign body lacerations in the duodenum could be considered an alternative to surgery if the patient remains stable without an acute abdomen. We demonstrated that a deep laceration site caused by the foreign body could close spontaneously upon removing the foreign body if no significant fibrosis has developed. While endoscopic closure of small bowel deep laceration is possible with the appropriate skills and devices, the need for surgical backup should always be available if endoscopic attempts were unsuccessful.

## Disclosure

*All authors disclosed no financial relationships.*

## Supplementary data

Video 1 The endoscope was advanced under direct visualization to the third part of the duodenum. A foreign body consistent with a ballpoint pen was identified in the duodenum. The sharp end of the pen formed a deep laceration through the second portion of the duodenum. The blunt end of the pen was ulcerated into the third portion of the duodenum with 2 additional pressure ulcers located in close proximity. Removal of the pen was first attempted using a snare without success. The foreign body was then successfully removed with a rat tooth. The second portion of the duodenum showed minimal oozing with contained deep laceration. The third portion of the duodenum showed 2 pressure ulcers.
